# Hypervirulence and Multiresistance to Antibiotics in *Klebsiella pneumoniae* Strains Isolated from Patients with Hospital- and Community-Acquired Infections in a Mexican Medical Center

**DOI:** 10.3390/microorganisms10102043

**Published:** 2022-10-16

**Authors:** Areli Bautista-Cerón, Eric Monroy-Pérez, Luis Rey García-Cortés, Ernesto Arturo Rojas-Jiménez, Felipe Vaca-Paniagua, Gloria Luz Paniagua-Contreras

**Affiliations:** 1 Facultad de Estudios Superiores Iztacala, Universidad Nacional Autónoma de México, Los Reyes Iztacala, Tlalnepantla 54090, Mexico; 2 Instituto Mexicano del Seguro Social, Naucalpan de Juárez 53370, Mexico; 3 Unidad de Biomedicina, Facultad de Estudios Superiores Iztacala, Universidad Nacional Autónoma de México, UNAM, Tlalnepantla 54090, Mexico; 4 Laboratorio Nacional en Salud, Diagnóstico Molecular y Efecto Ambiental en Enfermedades Crónico-Degenerativas, Facultad de Estudios Superiores Iztacala, Universidad Nacional Autónoma de México, Tlalnepantla 54090, Mexico; 5 Subdirección de Investigación Básica, Instituto Nacional de Cancerología, CDMX, Mexico City 14160, Mexico

**Keywords:** virulence genes, hypermucoviscous phenotype, serotypes, biofilm-forming, multidrug resistance

## Abstract

*Klebsiella pneumoniae* is a pathogenic bacterium associated with different infectious diseases. This study aimed to establish the different association profiles of virulence genes related to the hypermucoviscous phenotype (HM), capsular serotypes, biofilm formation, and multidrug resistance in *K. pneumoniae* strains from patients with hospital- and community-acquired infections. *K. pneumoniae* virulence genes and capsular serotypes were identified by PCR, antibiotic susceptibility by the Kirby–Bauer method, HM by the string test, and biofilm formation by measurement in polystyrene microtiter plates. Of a total of 150 strains from patients with hospital- (*n* = 25) and community-acquired infections (*n* = 125), 53.3% (80/150) were HM-positive and 46.7% (70/150) were HM-negative. HM-positive (68/80) and HM-negative (67/70) strains were biofilm-forming. Moreover, 58.7% (47/80) HM-positive and 57.1% (40/70) HM-negative strains were multidrug-resistant. Among HM-positive, HM-negative, and serotypes K1 (25/150), K2 (48/150), and non-K1/K2 strains, (77/150) the frequently detected adhesion genes were *fimH*, *mrkD*, *ycfM*, and *kpn*; *entB*, *irp2*, *irp1*, and *ybtS*, for iron acquisition; and *rmpA* for protectins. The gene association pattern *fimH/kpn/mrkD/ycfM/entB/irp1/irp2/ybtS/fyuA* (18/150) was frequent among the strains. *K. pneumoniae* strains from patients with hospital- and community-acquired infections demonstrated a wide diversity of virulence gene profiles related to phenotype (hypermucoviscosity, multidrug resistance, and biofilm formation) and serotypes.

## 1. Introduction

*Klebsiella pneumoniae* is a pathogenic bacterium associated with hospital-acquired infections [1] such as pneumonia, bacteremia, meningitis, liver abscesses, and surgical wound infections [2,3,4]; it also causes chronic community-acquired urinary tract infections [5]. The pathogenicity of *K. pneumoniae* is the result of a combination of different virulence factors such as adhesins (*fimH-1*, *mrkD*, *kpn*, and *ycfM*) involved in epithelial cell adhesion and biofilm formation; iron-acquisition systems (*entB*, *iutA*, *irp1*, *irp2*, *ybtS*, *fyuA*, and *iroN*); protectins (*magA* and *rmpA*); and toxins (*hlyA* and *cnf-1*) [6]. The emergence of hypervirulent variants of *K. pneumoniae* (hvKpn) with a hypermucoviscous phenotype (HM) has spread worldwide [7]. Several virulence factors have been associated with hvKpn strains, including the capsular serotypes K1 and K2, and the mucoviscosity-associated gene A (*magA*) and regulator of mucoid phenotype A (*rmpA*) genes [8]. The emergence of multidrug-resistant (MDR) and hvKpn strains represents a serious health problem that reduces therapeutic options for the treatment of infections [9], and increases morbidity and mortality [10]. Information regarding the epidemiology of *K. pneumonie* in Mexico is scarce. In a multicenter study involving 14 hospitals across 8 states between 2005 and 2012, the prevalence of hospital strains of *K. pneumoniae* that produced ESBLs (extended-spectrum lactamases) was 27.5% (299/1084) [11]. In 2014-2015, *K. pneumoniae* was found in 6.4% (94/1461) of hospitalized and outpatients with urinary tract infections at the Centro Médico Nacional de Occidente from Jalisco, Mexico [12]. According to a national report from 12 hospitals located in 5 states and Mexico City, in 2016-2017, it was reported that the frequency of *K. pneumoniae* in patients with bacteremia was 21.9% (699/3182) and in those with urinary tract infections was 8.5% (740/8718) [13]. However, the virulence genotype and phenotype profiles of hvKpn and MDR *K. pneumoniae*, and their involvement in the pathogenesis of different infectious diseases, have been poorly studied. It is for this reason that the current study established the different association profiles of virulence genes linked to HM, capsular serotypes, biofilm formation, and multidrug resistance in *K. pneumoniae* strains isolated from patients with hospital- and community-acquired infections.

## 2. Materials and Methods

### 2.1. Bacterial Strains

This study analyzed 150 strains of *K. pneumoniae* (one isolate per patient) collected between September 2019 and March 2020 at the microbiology laboratory of Hospital General Regional No. 72 (Instituto Mexicano del Seguro Social) located in the municipality of Tlalnepantla de Baz, Edo. de México, México. The strains were isolated from samples of patients with hospital-acquired infections, including bacteremia (*n* = 21), and pneumonia (*n* = 4), and from community-acquired infections including urinary tract infections (UTI; *n* = 61), respiratory infections (*n* = 53), infected ulcers (*n* = 8), and others (stool culture (*n* = 2) and tumor biopsy (*n* = 1)).

### 2.2. DNA Extraction and Identification of K. pneumoniae

DNA was extracted by the boiling method, as previously described [14]. *K. pneumoniae* was identified by polymerase chain reaction (PCR) based on the 16S-23S rDNA internal transcribed spacer [15]. The PCR assay was performed using 20 μL of reaction mixture that included: 12 μL of Taq DNA Polymerase 2X Master Mix RED (AMPLIQON, Copenhagen, Denmark’s), 1 μL of forward primer and 1 μL of reverse primer (10 pmol, Integrated DNA Technologies, San Diego, CA, USA), 3 μL of nuclease-free water, and 3 μL of DNA template (100 ng). *K. pneumoniae* ATCC 700,721 was used as a control in each assay.

### 2.3. Detection of Hypermucoviscosity

*K. pneumoniae* strains were grown on 5% ram’s blood agar (DIBICO, Edo. de México, México) overnight at 37 °C. Using a standard bacterial inoculation loop, the surface of individual colonies was touched to observe the formation of viscous strings. Strains were classified as HM-positive when viscous strings greater than 5 mm in length were produced [16].

### 2.4. Antibiotic Susceptibility

Antibiotic susceptibility was determined using the standard Kirby–Bauer disk diffusion method (Investigación Diagnóstica, CDMX, México). The following 12 antibiotics were tested: ampicillin (AM; 10 μg), carbenicillin (CB; 100 μg), cephalothin (CF; 30 μg), cefotaxime (CFX; 30 μg), ciprofloxacin (CPF; 5 μg), chloramphenicol (CL; 30 μg), nitrofurantoin (NF; 300 μg), amikacin (AK; 30 μg), gentamicin (GE; 10 μg), netilmicin (NET; 30 μg), norfloxacin (NOF; 10 μg), and trimethoprim with sulfamethoxazole (SXT; 25 μg). *Escherichia coli* ATCC 25,922 was used as a control in each assay. Results were interpreted using the Clinical and Laboratory Standards Institute guidelines [17].

### 2.5. Biofilm Formation Test

Biofilm quantification in *K. pneumoniae* strains was performed using polystyrene microtiter plates, as previously described [18]. *K. pneumoniae strains* were grown in Luria-Bertani broth for 18 h at 37 °C. The optical density (OD) was adjusted to 0.56 (2 × 10^7^ CFU/mL) at 540 nm. Culture aliquots (200 μL) were transferred to 96-well polystyrene microtiter plates and incubated for 24 h at 25 °C. A solution of 1% crystal violet (25 μL) was added to each well, and the plate was shaken and incubated at 25 °C for 15 min. The absorbance was measured on a Multiskan Ascent ELISA reader (Thermo Fisher Scientific) at 590 nm. The strains were classified as strong (OD > 0.500), moderate (0.500 < OD > 0.100), and weak (OD < 0.100) biofilm-producing.

### 2.6. Identification of Capsular Types K1 and K2

The primers and PCR conditions used to identify the K1 and K2 capsular serotypes were as previously described [19]. Each separate uniplex PCR assay was performed using 20 μL of reaction mix that included: 12 μL of Taq DNA Polymerase 2X Master Mix RED (AMPLIQON, Odense, Denmark), 1 μL of forward primer and 1 μL of reverse primer (10 pmol, Integrated DNA Technologies), 3 μL of nuclease-free water, and 3 μL of DNA template (100 ng).

### 2.7. Identification of Virulence Genes

The primers and PCR conditions used to assess the prevalence of virulence genes in *K. pneumoniae* strains were as previously described [6]. The following virulence factors were assessed: adhesins (*fimH-1* (type 1 fimbriae)*, mrkD* (type 3 fimbriae), *kpn* (fimH-like adhesin), and *ycfM* (outer membrane lipoprotein)), iron-acquisition systems (*entB* (enterobactin biosynthesis), *iutA* (aerobactin receptor), *irp1*, *irp2*, *ybtS (*yersiniabactin biosynthesis), *fyuA* (yersiniabactin receptor), and *iroN* (catecholate siderophores receptor)), protectins (*magA* (mucoviscosity-associated gene A) and *rmpA* (regulator of mucoid phenotype A)), and toxins (*hlyA* (hemolysin) and *cnf-1* (cytotoxic necrotizing factor 1)).

A χ^2^ test using the statistical program SPSS (*p* < 0.05) was applied to establish the differences between the frequency of virulence genes linked to the HM, capsular serotypes, biofilm formation, and antibiotic multiresistance in *K. pneumoniae* strains.

### 2.8. Unsupervised Hierarchical Clustering

*K. pneumoniae* strains were systematically grouped according to genotype, phenotype, serotype, and clinical origin from patients with hospital- and community-acquired infections using unsupervised hierarchical clustering with Gower’s similarity algorithm for categorical variables [20]. A categorical data matrix that included virulence genes, phenotype (hypermucoviscosity, biofilm formation, and multidrug resistance), capsular serotypes, and the clinical origin of patients with hospital-acquired infections (bacteremia and pneumonia) and community-acquired infections (UTIs, respiratory infections, infected ulcers, and others) was created in R (v3.6.1) using the cluster package (2.1.0). The distance of each strain was calculated based on the overall similarity coefficient, which estimates the maximum possible absolute discrepancy between each combined pair of strains. With the calculated distances, mutually exclusive groups were clustered by the Ward’s method using R [21]. Strains were visualized in a genotype–phenotype–serotype distribution diagram with a dendrogram constructed using hclust (v3.6.2, R core).

## 3. Results

### 3.1. Origin of Strains and Distribution of Virulence Genes and Serotypes

*K. pneumoniae* strains were obtained from specimens of patients with community-acquired ((UTIs (*n* = 61; Table 1), and respiratory infections (*n* = 53)) as well as hospital-acquired infections (bacteraemia; *n* = 21). The frequently identified genes in these strains were *fimH*, *mrkD*, *ycfM*, and *kpn* (coding for adhesins), *entB*, *irp2*, *irp1*, and *ybtS* (iron uptake systems), and *rmpA* (protectins) (Table 1). Notably, 16.6% (25/150) of strains belonged to serotype K1, and 32% (48/150) to K2. No statistically significant differences in frequency depending on clinical origin from patients with hospital- and community-acquired infections were found between *fimH*, *mrkD*, *ycfM*, *irp1*, *irp2*, *ybtS*, *fyuA*, *iroN*, *hlyA*, and *cnf-1* genes (*p* < 0.05, Table 1). However, the detection rates of *kpn*, *entB*, *iutA*, *magA*, and *rmpA* genes, and K1, K2, and non-K1/K2 serotypes differed depending on the clinical origin from patients with hospital- and community-acquired infections (*p* < 0.05).

### 3.2. Virulence Gene Association Patterns

We identified 86 distinct virulence gene association patterns (Table 2). The distribution of virulence patterns was similar among strains of different clinical origin from patients with hospital- and community-acquired infections (*p* < 0.05, Table 2). The exceptions were patterns no. 1 (*n* = 18), frequently associated with strains linked to bacteremia and infected ulcers; no. 3 and no. 20, associated with others (stool culture (*n* = 2) and tumor biopsy (*n* = 1)); and no. 18, associated with pneumonia (*p* < 0.05).

### 3.3. Hypermucoviscosity and Multiresistance to Antibiotics

Our hypermucoviscosity evaluation showed that 53.3% (80/150) of the strains were HM-positive (hospital-acquired (*n =* 8) and community-acquired (*n =* 72) and 46.7% (70/150) were HM-negative (hospital-acquired (*n =* 17) and community-acquired (*n =* 53)) (Table 3). HM-positive and HM-negative *K. pneumoniae* strains isolated from patients with hospital- and community-acquired infections had high percentages of resistance for the beta-lactams AM, CB, and CF. There was a significant difference in the percentages of resistance to seven antibiotics (CF, CFX, CPF, GE, NET, NOF, and SXT) among strains isolated from patients with hospital- and community-acquired infections (*p* < 0.05, Table 3). For these antibiotics, the percentages were higher for hospital-acquired strains (HM-positive and HM-negative), compared with community-acquired strains (HM-positive and HM-negative). In the same way, the percentages of multiresistant strains against 10–12 antimicrobials were higher in hospital-acquired strains ((HM-positive (5/8) and HM-negative (6/17)) compared with community-acquired strains (HM-positive (9/72) and HM -negative (7/53)).

### 3.4. Biofilm Formation

Biofilm formation was observed in 85% (68/80) of the HM-positive strains (Table 4) and in 95.7% (67/70) of the HM-negative strains (Table 5). The frequency of strong biofilm formation was similar between HM-positive (11/68; Table 4) and HM-negative (11/67; Table 5) strains; moderate and weak biofilm formation revealed the highest rate in HM-negative (29/67; Table 5) and HM-positive strains (39/68; Table 4), respectively. The frequency and distribution of virulence genes and serotypes K1, K2, and non-K1/K2 was similar between strains (independent of biofilm formation capacity, HM phenotype presentation, and hospital- and community-acquired infections) (*p* < 0.05, Table 4 and Table 5).

### 3.5. Distribution of Virulence Genes Related to Serotypes and Hypermucoviscosity

In HM-positive strains, gene frequency was consistent between serotypes K1 (exclusively in community-acquired), K2 (hospital- and community-acquired), and non-K1/K2 (hospital- and community-acquired; *p* < 0.05, Table 6). Significant differences were also found in HM-negative strains (hospital- and community-acquired) in *irp1*, *ybtS*, *fyuA*, *magA*, and *rmpA* genes according to the capsular serotype (*p* < 0.05, Table 7). The *magA* gene was identified in 100% of community-acquired HM-positive (*n* = 18; Table 6) or HM-negative (*n* = 7; Table 7) strains associated with serotype K1. The *magA+rmpA+* gene association was detected in serotype K1 of community-acquired HM-positive (16/18; Table 6) and HM-negative (6/7; Table 7) strains.

### 3.6. Genotypic and Phenotypic Diversity

**Figure 1:**
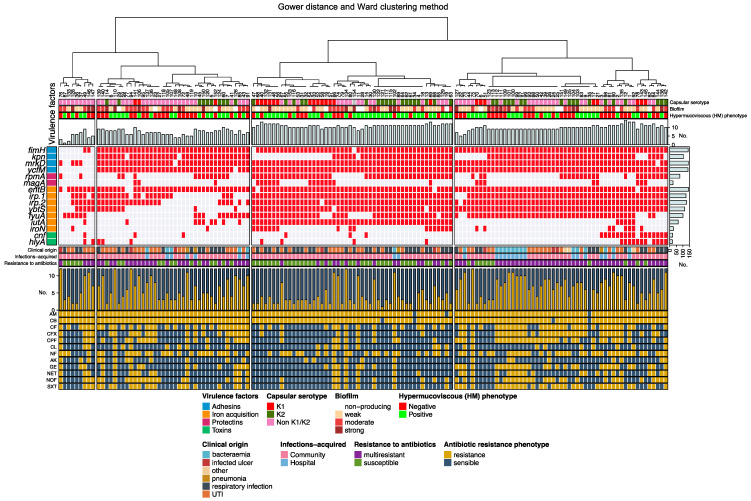
Clustering of *K. pneumoniae* strains according to virulence genotype profile and its association with clinical origin from patients with hospital- and community-acquired infections, capsular serotype, biofilm formation, hypermucoviscous phenotype, and antibiotic resistance. Positivity and negativity for a given genotype is represented by a red and grey rectangle, respectively. Cladograms of the strains are shown at the top. Upper axis: identification number of the strains. Upper left axis: virulence genes. Lower left axis: antibiotic resistance (resistance to a given antibiotic is represented in yellow and susceptibility in blue). Upper bars (grey): frequency of the different virulence genes detected in each strain. Right bars (grey): frequency of each virulence gene. Lower bars (yellow): frequency of antibiotic resistance detected in each strain. Other: stool culture (*n* = 2) and tumor biopsy (*n* = 1).

Three major groups were identified based on the similarities between *K. pneumoniae* strains (Figure 1). Group 1 was divided into several subgroups and consisted of 47 strains (range of number of strains from 76 to 91); groups 2 and 3 consisted of 50 (47 to 63) and 53 (127 to 142) strains, respectively. In all three groups, we identified pairs of strains of patients with community-acquired infections: 139 (UTI) and 140 (UTI); 117 (respiratory infections) and 121 (UTI); 17 (respiratory infections) and 21 (respiratory infections) with the same genotype, phenotype, and serotype (100% similarity; Figure 1), and strains (range 123 to 25) with the same genotype, but with different serotype, phenotype (HM, biofilm formation, and multiresistance to antibiotics) and clinical origin of patients with hospital-acquired infections (bacteremia (*n* = 8)) and community-acquired infections (UTIs (*n* = 8) and infected ulcers (*n* = 2)) (Figure 1).

## 4. Discussion

The results demonstrated that *K. pneumoniae* is an important pathogen causing hospital-acquired and community-acquired infections. *K. pneumoniae* has been reported to account for 2–7% of community-acquired UTIs [22,23], while pneumonia comprises 22–23% of cases requiring intensive care unit admission [24]. An extremely serious consequence of *K. pneumoniae* pneumonias and UTIs is their subsequent spread to the blood, causing bacteremia [25].

The pathogenicity of *K. pneumoniae* results from the different innate virulence factors, such as adhesins that promote colonization and tissue invasion. The distribution of adhesin genes (*fimH*, *mrkD*, and *ycfM*) was similar among strains of different clinical origin from patients with hospital- and community-acquired infections, which was consistent with the findings of a previous report on *K. pneumoniae* isolates from urine, blood, pus, and lungs [6]. The high frequency of *fimH* (94%) and *mrkD* (95.3%) in our strains, which code for fimbria types 1 and 3, respectively, demonstrates their virulence, since fimbria type 1 is an important marker in UTIs [26], and type 3 mediates adhesion to kidney, lung, and bladder tissue [27].

Iron facilitates a large number of cellular activities essential for bacterial survival and reproduction [28]. The overall prevalence of iron uptake genes among strains of different origin from patients with hospital- and community-acquired infections was similar in this study, with *entB* (96%), *irp2* (86%), *irp1* (83.3%), and *ybtS* (76.6%) showing percentages higher than those described in *K. pneumoniae* strains isolated from different specimens [6] or from renal transplant and non-transplant patients [29].

The overall frequency of the protectin genes *magA* (16.6%) and *rmpA* (52%) and the capsular serotypes K1 (16.6%) and K2 (32%) was significantly different among *K. pneumoniae* strains of different clinical origin from patients with hospital- and community-acquired infections. *magA* and serotype K1 were frequently identified in strains associated with respiratory infections and infected ulcers; *rmpA*, with respiratory infections and others (stool culture and tumor biopsy); and serotype K2 (32%), with respiratory infections and bacteremia. The frequencies of virulence markers *magA* and *rmpA*, and serotypes K1 and K2 found in this study are similar to those described in *K. pneumoniae* strains isolated from pneumonia and UTI [30], and lower than those described in strains isolated from pyogenic liver abscesses [31].

The overall frequencies of *hlyA* (13.3%) and *cnf-1* (14.6%) were similar among strains from patients with hospital- and community-acquired infections and higher than those described in *K. pneumoniae* strains isolated from different specimens [6,29]. Toxin HlyA causes tissue damage, favoring invasion and nutrient release from the host [32], while CNF1 has been implicated in kidney invasion [33].

A wide distribution of virulence marker association patterns was identified in *K. pneumoniae* strains from different clinical origins from patients with hospital- and community-acquired infections. This suggests that different virulence gene expression profiles may exist over the course of different infections, increasing infection severity, especially in immunocompromised patients.

Multidrug-resistance frequency was high among the hospital-acquired strains and community-acquired strains. The high resistance to beta-lactam antibiotics may be due to the marked increase in the number of *K. pneumoniae* strains producing extended-spectrum beta-lactamases [34]. During the COVID-19 pandemic, a study conducted in St. Andrea Hospital, Rome, found an increase in extended-spectrum beta-lactamase-producing *K. pneumoniae* strains in the COVID-19 department, compared with other medical departments [35]. The percentage of resistance to AM, NF, AK, and GE found in hospital- and community-acquired HM-positive and HM-negative strains was similar to that described in *K. pneumoniae* strains isolated from pneumonia patients with and without diabetes [36], and higher than those found for CFX, CPF, and AM in HM-positive (*n* = 10) and HM-negative (*n* = 71) strains isolated from urine [37].

HM-positive (85%) and HM-negative (95.7%) hospital- and community-acquired *K. pneumoniae* strains in this study were biofilm-forming. No significant differences were found in the three categories of biofilm formation (weak, moderate, and strong) in HM-positive and HM-negative strains isolated from patients with hospital- and community acquired infections. The percentages of weak, moderate, and strong biofilm formation of HM-positive and HM-negative strains are higher than those described in *K. pneumoniae* strains isolated from different clinical origins [38,39]. The high percentage of biofilm formation among HM-positive and HM-negative strains isolated from patients with hospital- and community-acquired infections demonstrates their ability to cause acute infections, since biofilms protect bacteria against the host immune response and antimicrobials [40]. Notably, *mrkD* (type 3 fimbria), involved in biofilm formation in *K. pneumoniae* [41] was frequently detected among biofilm-forming HM-positive and HM-negative strains.

In HM-positive strains, the distribution of virulence markers associated with serotypes K2 and non-KI/K2 was similar among hospital- and community-acquired strains. With respect to HM-negative strains, *irp1*, *ybtS*, and *fyuA* were more prevalent in K2 of the hospital-acquired strains, and *magA* and *rmpA* in K1 of the community-acquired strains, compared with the other serotypes. As for HM-positive strains exclusively acquired in the community, 88.9% were *rmpA+/K1* and 91.7% were *rmpA+/K2*. The *magA* marker, which is specific to serotype K1, was detected in 100% of HM-positive K1-associated community-acquired strains. *rmpA* and *rmpA2* have been found in the pLVPK plasmid (219 KB) of hvKpn/HM-positive strains, along with other virulence factors encoding for several siderophore systems and genes associated with resistance to tellurite, copper, silver, and lead [42]. The frequencies of *rmpA+*/*HM+/K1* and *rmpA+*/*HM+/K2* detected in our *K. pneumoniae* hospital- and community-acquired strains and reported in strains from patients with liver abscesses [19] are higher than those described in Asia in HM-positive *K. pneumoniae* strains associated with pneumonia and UTIs [30,37].

Unsupervised hierarchical clustering analysis allowed the grouping of hospital- or community-acquired strains with the same genotype, phenotype, and serotype. Such is the case of community-acquired strains 139 (respiratory infection) and 140 (UTI), which presented the same genotype (*fimH/kpn/mrkD/ycfM/entB/irp1/irp2/ybtS*) and phenotype (HM-positive, moderate biofilm formation, and multidrug resistance), and were both non-K1/K2. Similarly, some community-acquired strains (17, 21, 117, and 121) also shared the same genotype, phenotype, and serotype, as was found in strains 83 (UTI) and 9 (bacteremia) acquired in the community and in the hospital, respectively. These results indicate that these *K. pneumoniae* strains could have been acquired by different patients in the community and some of these community strains may already be present in the hospital. This analysis demonstrates the wide distribution and versatility of the genotype–phenotype–serotype of strains related to different clinical origins of patients with hospital- and community-acquired infections, showing their ability to cause acute and fatal infections, especially those that present virulence factors characteristic of hvKpn strains described in other regions [43].

## 5. Conclusions

A global analysis of the systematic clustering of virulence genotype profiles related to HM, biofilm formation, capsular serotypes, and multidrug resistance in *K. pneumoniae* strains from different clinical origins of patients with hospital- and community-acquired infections has not been previously reported. Therefore, our findings are relevant and may help to better understand the role and pathogenesis of *K. pneumoniae* during infections, and guide treatment strategies against this important opportunistic pathogen.

## Figures and Tables

**Table 1:** Frequency of virulence genes, and serotypes in strains of *K. pneumoniae* isolated from patients with hospital- and community-acquired infections.

Function	Gene or Capsular Serotype	Origin of the Strains						*p*-Value	Total (*n* = 150)
		Hospital-Acquired (*n* = 25)		Acquired in the Community (*n* = 125)					
		Bacteremia *n* = 21 (%)	Pneumonia *n* = 4 (%)	UTI *n* = 61 (%)	Respiratory Infection *n* = 53 (%)	Infected Ulcer *n* = 8 (%)	*Other *n* = 3 (%)		
Adhesins	*fimH*	21 (100)	4 (100)	57 (93.4)	49 (92.5)	7 (87.5)	3 (100)	0.754	141
	*mrkD*	21 (100)	4 (100)	57 (93.4)	51 (96.2)	7 (87.5)	3 (100)	0.690	143
	*kpn*	20 (95.2)	4 (100)	42 (68.9)	32 (60.4)	5 (62.5)	3 (100)	0.033	106
	*ycfM*	21 (100)	4 (100)	55 (90.2)	50 (94.3)	7 (87.5)	3 (100)	0.622	140
Iron-acquisition systems	*entB*	21 (100)	4 (100)	60 (98.4)	50 (94.3)	6 (75)	3 (100)	0.028	144
	*irp1*	18 (85.7)	2 (50)	52 (85.2)	45 (84.9)	5 (62.5)	3 (100)	0.248	125
	*irp2*	19 (90.5)	2 (50)	54 (88.5)	46 (86.8)	5 (62.5)	3 (100)	0.102	129
	*ybtS*	16 (76.2)	3 (75)	46 (75.4)	42 (79.2)	5 (62.5)	3 (100)	0.839	115
	*fyuA*	17 (81)	3 (75)	38 (62.3)	37 (69.8)	4 (50)	3 (100)	0.379	102
	*iutA*	3 (14.3)	2 (50)	22 (36.1)	34 (64.2)	2 (25)	1(33.3)	0.004	64
	*iroN*	2 (9.5)	0	9 (14.8)	13 (24.5)	2 (25)	1(33.3)	0.465	27
Protectins	*magA*	0	0	8 (13.1)	15 (28.3)	2 (25)	0	0.038	25
	*rmpA*	4 (19)	2 (50)	23 (37.7)	43 (81.1)	4 (50)	2 (66.7)	0.0004	78
Toxins	*hlyA*	1 (4.8)	0	8 (13.1)	9 (17)	1 (12.5)	1 (33.3)	0.607	20
	*cnf-1*	3 (14.3)	1(25)	9 (14.8)	8 (15.1)	0	1 (33.3)	0.767	22
Capsular serotype	K1	0	0	8 (13.1)	15 (28.3)	2 (25)	0	0.03	25
	K2	11 (52.4)	0	10 (16.4)	25 (47.2)	1 (12.5)	1 (33.3)	0.001	48
	Non-K1/K2	10 (47.6)	4 (100)	43 (70.5)	13 (24.1)	5 (62.5)	2 (66.7)	0.0001	77

Notes: significant *p*-values (<0.05) are presented in bold. * Other: stool culture (*n* = 2) and tumor biopsy (*n* = 1).

**Table 2:** Distribution of virulence gene association patterns in strains of *K. pneumoniae* isolated from patients with hospital- and community-acquired infections.

Function	Gene or Capsular Serotype	Origin of the Strains						*p*-Value	Total (*n* = 150)
		Hospital-Acquired (*n* = 25)		Acquired in the Community (*n* = 125)					
		Bacteremia *n* = 21 (%)	Pneumonia *n* = 4 (%)	UTI *n* = 61 (%)	Respiratory Infection *n* = 53 (%)	Infected Ulcer *n* = 8 (%)	* Other *n* = 3 (%)		
No.	Patterns of Virulence Genes								Total (*n* = 150)
1	*fimH/kpn/mrkD/ycfM/entB/irp1/irp2/ybtS/fyuA*	8 (38.1)	0	8 (13.1)	0	2 (25)	0	0.0001	18
2	*fimH/kpn/mrkD/ycfM/rmpA/entB/irp1/irp2/ybtS/fyuA/iutA*	1 (4.8)	1 (25)	4 (6.5)	7 (13.2)	0	0	0.458	13
3	*fimH/kpn/mrkD/ycfM/rmpA/entB/irp1/irp2/ybtS/fyuA*	3 (14.3)	0	2 (3.3)	1 (1.9)	0	2 (66.7)	0.006	8
4	*fimH/mrkD/ycfM/rmpA/entB/irp1/irp2/ybtS/fyuA/iutA*	0	0	2 (3.3)	3 (5.7)	0	0	0.826	5
5	*fimH/mrkD/ycfM/rmpA/magA/entB/irp1/irp2/ybtS/fyuA/iutA/iroN*	0	0	1 (1.6)	3 (5.7)	0	0	0.593	4
6	*fimH/kpn/mrkD/ycfM/entB/irp1/irp2*	1 (4.8)	0	3 (4.9)	0	0	0	0.484	4
7	*fimH/kpn/mrkD/ycfM/entB/irp1/irp2/ybtS*	0	0	3 (4.9)	0	0	0	0.478	3
8	*fimH/kpn/mrkD/ycfM/entB/irp1*	2 (9.5)	0	0	1 (1.9)	0	0	0.198	3
9	*fimH/mrkD/ycfM/rmpA/magA/entB/irp1/irp2/ybtS/fyuA/iutA*	0	0	1 (1.6)	2 (3.8)	0	0	0.490	3
10	*fimH/kpn/mrkD/ycfM/rmpA/entB/irp1/irp2/ybtS/fyuA/iutA/iroN*	0	0	0	3 (5.7)	0	0	0.314	3
11	*fimH/kpn/mrkD/ycfM/entB/irp1/irp2/ybtS/hlyA*	0	0	1 (1.6)	1 (1.9)	0	0	1	2
12	*fimH/kpn/mrkD/ycfM/rmpA/entB/irp1/irp2/ybtS*	0	0	1 (1.6)	1 (1.9)	0	0	1	2
13	*fimH/kpn/mrkD/ycfM/entB*	0	1 (25)	0	1 (1.9)	0	0	0.106	2
14	*fimH/kpn/mrkD/ycfM/rmpA/entB/fyuA/iutA*	0	0	0	2 (3.8)	0	0	0.546	2
15	*fimH/mrkD/ycfM/rmpA/magA/entB/irp1/irp2/ybtS/iutA*	0	0	1 (1.6)	1 (1.9)	0	0	1	2
16	*fimH/kpn/mrkD/ycfM/entB/irp1/irp2/ybtS/fyuA/iutA*	0	0	1 (1.6)	1 (1.9)	0	0	1	2
17	*fimH/kpn/mrkD/ycfM/rmpA/magA/entB/irp1/irp2/ybtS/fyuA*	0	0	0	2 (3.8)	0	0	0.546	2
18	*fimH/kpn/mrkD/ycfM/entB/irp1/irp2/ybtS/fyuA/cnf-1*	1 (4.8)	1 (25)	0	0	0	0	0.022	2
19	*fimH/kpn/mrkD/ycfM/entB/irp1/irp2/ybtS/fyuA/iroN/cnf-1*	1 (4.8)	0	1 (1.6)	0	0	0	0.423	2
20	*fimH/kpn/mrkD/ycfM/entB/irp1/irp2/ybtS/fyuA/iutA/iroN/cnf-1/hlyA*	1 (4.8)	0	0	0	0	1 (33.3)	0.015	2
21-86	Distinct patterns	3 (14.3)	1 (25)	32 (52.4)	24 (45.3)	6 (75)	0		66

Notes: significant *p*-values (<0.05) are presented in bold. * Other: stool culture (*n* = 2) and tumor biopsy (*n* = 1).

**Table 3:** Resistance and multiresistance to antibiotics according to hypermucoviscous phenotype (HM) in strains of *K. pneumoniae* isolated from patients with hospital- and community-acquired infections.

Antibiotics	Hypermucoviscous (HM) Phenotype (*n* = 150)				
	Hospital-Acquired (*n* = 25)		Acquired in the Community (*n* = 125)		
	HM-Positive (*n* = 8) %	HM-Negative (*n* = 17) %	HM-Positive (*n* = 72) %	HM-Negative (*n* = 53) %	*p*-Value
Ampicillin	8 (100)	17 (100)	70 (97.2)	53 (100)	1
Carbenicillin	8 (100)	17 (100)	70 (97.2)	53 (100)	1
Cephalothin	6 (75)	17 (100)	35 (48.6)	23 (43.4)	**0.00004**
Cefotaxime	7 (87.5)	16 (94.1)	20 (27.8)	23 (43.4)	**0.000001**
Ciprofloxacin	7 (87.5)	16 (94.1)	21 (29.2)	20 (37.7)	**0.0000006**
Chloramphenicol	1 (12.5)	4 (23.5)	18 (25)	8 (15.1)	0.41
Nitrofurantoin	7 (87.5)	10 (58.8)	36 (50)	33 (62.3)	0.507
Amikacin	3 (37.5)	4 (23.5)	12 (16.7)	9 (17)	0.772
Gentamicin	5 (62.5)	15 (88.2)	20 (27.7)	43 (81.1)	**0.0000006**
Netilmicin	5 (62.5)	10 (58.8)	14 (19.4)	13 (24.5)	**0.0007**
Norfloxacin	6 (75)	11(64.7)	19 (26.4)	25 (47.2)	**0.011**
Trimethoprim-sulfamethoxazole	5 (62.5)	16 (94.1)	22 (30.6)	24 (45.3)	**0.00001**
Multiresistance (different families of antibiotics; *n* = 86)					
4-6	2 (25)	1 (5.9)	10 (13.9)	14 (26.4)	
7-9	1 (12.5)	2 (11.8)	20 (27.8)	10 (18.9)	
10-12	5 (62.5)	6 (35.3)	9 (12.5)	7 (13.2)	**0.00006**

Notes: significant *p*-values (<0.05) are presented in bold.

**Table 4:** Distribution of virulence genes according to hypermucoviscous (HM)-positive strains and biofilm formation of *K. pneumoniae* isolated from patients with hospital- and community-acquired infections.

Function	Gene or Capsular Serotype	Hypermucoviscous (HM)-Positive (*n* = 80)								
		Biofilm (+) (*n* = 68)						Biofilm (−) (*n* = 12)		
		Weak (*n* = 39) %		Moderate (*n* = 18) %		Strong (*n* = 11) %		Non-Producing (*n* = 12) %		
		Hospital (*n* = 3)	Community (*n* = 36)	Hospital (*n* = 3)	Community (*n* = 15)	Hospital (*n* = 2)	Community (*n* = 9)	Hospital (*n* = 0)	Community (*n* = 12)	*p*-Value
Adhesins	*fimH*	3 (100)	35 (97.2)	3 (100)	14 (93.3)	2 (100)	7 (77.8)	0	12 (100)	0.152
	*mrkD*	3 (100)	35 (97.2)	3 (100)	13 (86.7)	2 (100)	9 (100)	0	12 (100)	0.322
	*kpn*	3 (100)	25 (69.4)	3 (100)	8 (56.3)	2 (100)	7 (77.8)	0	7 (58.3)	0.557
	*ycfM*	3 (100)	35 (97.2)	3 (100)	13 (86.7)	2 (100)	8 (88.9)	0	11 (91.7)	0.375
Iron-acquisition systems	*entB*	3 (100)	35 (97.2)	3 (100)	12 (80)	2 (100)	9 (100)	0	11 (91.7)	0.117
	*irp1*	3 (100)	32 (88.9)	3 (100)	12 (80)	2 (100)	9 (100)	0	11 (91.7)	0.625
	*irp2*	3 (100)	32 (88.9)	2 (66.7)	13 (86.7)	2 (100)	9 (100)	0	11 (91.7)	0.625
	*ybtS*	3 (100)	29 (80.6)	2 (66.7)	11 (73.3)	2 (100)	7 (77.8)	0	12 (100)	0.26
	*fyuA*	3 (100)	29 (80.6)	2 (66.7)	11 (73.3)	2 (100)	5 (55.6)	0	8 (66.7)	0.447
	*iutA*	0	19 (52.8)	1 (33.3)	10 (66.7)	0	5 (55.6)	0	7 (58.3)	0.790
	*iroN*	0	9 (25)	0	2 (13.3)	0	3 (33.3)	0	2 (16.7)	0.698
Protectins	*magA*	0	9 (25)	0	3 (20)	0	2 (22.2)	0	4 (33.3)	0.773
	*rmpA*	1 (33.3)	27 (75)	1 (33.3)	11 (73.3)	1 (50)	4 (44.4)	0	7 (58.3)	0.392
Toxins	*hlyA*	0	2 (5.6)	0	2 (13.3)	0	2 (22.2)	0	0	0.255
	*cnf*	0	3 (8.3)	0	1 (6.7)	0	1 (11.1)	0	2 (16.7)	0.750
Serotype	K1	0	9 (25)	0	3 (20)	0	2 (22.2)	0	4 (33.3)	0.773
	K2	2 (66.7)	15 (41.7)	0	4 (26.7)	2 (100)	3 (33.3)	0	2 (16.7)	0.19
	Non-K1/K2	1 (33.3)	12 (33.3)	3 (66.7)	8 (53.3)	0	4 (44.4)	0	6 (50)	0.228

**Table 5:** Distribution of virulence genes according to hypermucoviscous (HM)-negative strains and biofilm formation of *K. pneumoniae* isolated from patients with hospital- and community-acquired infections.

Function	Gene or Capsular Serotype	Hypermucoviscous (HM)-Negative (*n* = 70)								
		Biofilm (+) (*n* = 67)						Biofilm (−) (*n* = 3)		
		Weak (*n* = 27) %		Moderate (*n* = 29) %		Strong (*n* = 11) %		Non-Producing (*n* = 3) %		
		Hospital (*n* = 5)	Community (*n* = 22)	Hospital (*n* = 8)	Community (*n* = 21)	Hospital (*n* = 4)	Community (*n* = 7)	Hospital (*n* = 0)	Community (*n* = 3)	*p*-Value
Adhesins	*fimH*	5 (100)	19 (86.4)	8 (100)	20 (95.2)	4 (100)	6 (85.7)	0	3 (100)	0.576
	*mrkD*	5 (100)	20 (90.9)	8 (100)	21 (100)	4 (100)	5 (71.4)	0	3 (100)	0.117
	*kpn*	4 (80)	11 (50)	8 (100)	18 (85.7)	4 (100)	3 (42.9)	0	3 (100)	0.015
	*ycfM*	5 (100)	20 (90.9)	8 (100)	20 (95.2)	4 (100)	5 (71.4)	0	3 (100)	0.396
Iron-acquisition systems	*entB*	5 (100)	22 (100)	8 (100)	20 (95.2)	4 (100)	7 (100)	0	3 (100)	1
	*irp1*	2 (40)	19 (86.4)	6 (75)	17 (81)	4 (100)	3 (42.9)	0	2 (66.7)	0.676
	*irp2*	3 (60)	18 (81.8)	7 (87.5)	18 (85.7)	4 (100)	5 (71.4)	0	2 (66.7)	0.67
	*ybtS*	4 (80)	17 (77.3)	4 (50)	14 (66.7)	4 (100)	5 (71.4)	0	1 (33.3)	0.224
	*fyuA*	3 (60)	13 (59.1)	6 (75)	12 (57.1)	4 (100)	3 (42.9)	0	1 (33.3)	0.873
	*iutA*	2 (40)	11 (50)	1 (12.5)	5 (23.8)	1 (25)	1 (14.3)	0	1 (33.3)	0.111
	*iroN*	1 (20)	7 (31.8)	1 (12.5)	2 (9.5)	0	0	0	0	0.093
Protectins	*magA*	0	4 (18.2)	0	2 (9.5)	0	0	0	1 (33.3)	0.214
	*rmpA*	1 (20)	13 (59.1)	0	7 (33.3)	2 (50)	1 (14.3)	0	2 (66.7)	0.096
Toxins	*hlyA*	1 (20)	6 (27.3)	0	6 (28.6)	0	1 (14.3)	0	0	0.717
	*cnf*	2 (40)	5 (22.7)	2 (25)	6 (28.6)	0	0	0	0	0.198
Serotype	K1	0	4 (18.2)	0	2 (9.5)	0	0	0	1 (33.3)	0.214
	K2	0	7 (31.8)	3 (37.5)	4 (19)	3 (75)	2 (28.6)	0	1 (33.3)	0.557
	Non-K1/K2	5 (100)	11 (50)	2 (25)	18 (85.7)	1 (25)	5 (71.4)	0	1 (33.3)	0.584

**Table 6:** Distribution of virulence genes according to hypermucoviscous (HM)-positive strains and capsular serotype of *K. pneumoniae* isolated from patients with hospital- and community-acquired infections.

Function	Gene	Hypermucoviscous (HM)-Positive (*n* = 80)						
		Capsular Serotype No. (%)						
		K1 (*n* = 18)		K2 (*n* = 28)		Non-K1/K2 (*n* = 34)		*p*-Value
		Hospital (*n* = 0)	Community (*n* = 18)	Hospital (*n* = 4)	Community (*n* = 24)	Hospital (*n* = 4)	Community (*n* = 30)	
Adhesins	*fimH*	0	18 (100)	4 (100)	23 (95.8)	4 (100)	27 (90)	0.540
	*mrkD*	0	18 (100)	4 (100)	24 (100)	4 (100)	27 (90)	0.238
	*kpn*	0	4 (22.2)	4 (100)	22 (91.7)	4 (100)	21 (70)	**0.000002**
	*ycfM*	0	18 (100)	4 (100)	23 (95.8)	4 (100)	26 (86.7)	0.280
Iron-acquisition systems	*entB*	0	16 (88.9)	4 (100)	23 (95.8)	4 (100)	28 (93.3)	0.715
	*irp1*	0	18 (100)	4 (100)	19 (79.2)	4 (100)	27 (90)	0.170
	*irp2*	0	18 (100)	4 (100)	20 (83.3)	3 (75)	27 (90)	0.280
	*ybtS*	0	17 (94.4)	4 (100)	18 (75)	3 (75)	24 (80)	0.308
	*fyuA*	0	14 (77.8)	4 (100)	18 (75)	3 (75)	21 (70)	0.797
	*iutA*	0	11 (61.1)	0	17 (70.8)	1 (25)	13 (43.3)	0.241
	*iroN*	0	8 (44.4)	0	4 (16.7)	0	4 (13.3)	**0.024**
Protectins	*magA*	0	18 (100)	0	0	0	0	**0.000002**
	*rmpA*	0	16 (88.9)	1 (25)	22 (91.7)	2 (50)	11 (36.7)	**0.00008**
Toxins	*hlyA*	0	1 (5.6)	0	2 (8.3)	0	3 (10)	1
	*cnf-1*	0	0	0	3 (12.5)	0	4 (13.3)	0.41

Notes: significant *p*-values (<0.05) are presented in bold.

**Table 7:** Distribution of virulence genes according to hypermucoviscous (HM)-negative strains and capsular serotype of *K. pneumoniae* isolated from patients with hospital- and community-acquired infections.

Function	Gene	Hypermucoviscous (HM)-Negative (*n* = 70)						
		Capsular Serotype No. (%)						
		K1 (*n* = 7)		K2 (*n* = 20)		Non-K1/K2 (*n* = 43)		*p*-Value
		Hospital (*n* = 0)	Community (*n* = 7)	Hospital (*n* = 7)	Community (*n* = 13)	Hospital (*n* = 10)	Community (*n* = 33)	
Adhesins	*fimH*	0	7 (100)	7 (100)	12 (92.3)	10 (100)	29 (87.9)	1
	*mrkD*	0	7 (100)	7 (100)	12 (92.3)	10 (100)	30 (90.9)	1
	*kpn*	0	3 (42.9)	7 (100)	9 (69.2)	9 (90)	23 (69.7)	0.256
	*ycfM*	0	7 (100)	7 (100)	13 (100)	10 (100)	28 (84.8)	0.219
Iron-acquisition systems	*entB*	0	7 (100)	7 (100)	13 (100)	10 (100)	32 (97)	1
	*irp1*	0	6 (85.7)	7 (100)	12 (92.3)	5 (50)	23 (69.7)	**0.042**
	*irp2*	0	5 (71.4)	7 (100)	12 (92.3)	7 (70)	26 (78.8)	0.082
	*ybtS*	0	4 (57.1)	7 (100)	11 (84.6)	5 (50)	22 (66.7)	**0.035**
	*fyuA*	0	2 (28.6)	7 (100)	10 (76.9)	6 (60)	17 (51.5)	**0.005**
	*iutA*	0	4 (57.1)	1 (14.3)	7 (53.8)	3 (30)	7 (21.2)	0.206
	*iroN*	0	2 (28.6)	1 (14.3)	3 (23.1)	1 (10)	4 (12.1)	0.443
Protectins	*magA*	0	7 (100)	0	0	0	0	**0.000000006**
	*rmpA*	0	6 (85.7)	2 (28.6)	11 (84.6)	1 (10)	6 (18.2)	**0.00004**
Toxins	*hlyA*	0	3 (42.9)	0	4 (30.8)	1 (10)	6 (18.2)	0.425
	*cnf-1*	0	3 (42.9)	2 (28.6)	3 (23.1)	2 (20)	5 (15.2)	0.395

Notes: significant *p*-values (<0.05) are presented in bold.

## Data Availability

Not applicable.
